# Preserving Soybean Oil for the Frying of Breaded Butterfly Shrimp Using Natural Rosemary Antioxidant

**DOI:** 10.1155/2023/5984636

**Published:** 2023-03-24

**Authors:** Chaimae Moufakkir, Yassine Kharbach, Mariam Tanghort, Abdelilah Dassouli, Adnane Remmal

**Affiliations:** ^1^Biotechnology Laboratory, Faculty of Science Dhar El Mahraz, Sidi Mohamed Ben Abdellah University, P.O. Box 1796, Fez 30050, Morocco; ^2^Laboratory of Applied Chemistry, Faculty of Sciences and Technology, Sidi Mohamed Ben Abdellah University, P.O. Box 2202, Fez M-30050, Morocco

## Abstract

Fried foods and frying oil are subjects that warrant the attention of researchers because of their high consumption. Indeed, frying conditions make these oils very sensitive to lipid oxidation which deteriorates the quality and nutritional properties of the food. In this study, we examined the effect of rosemary extract (ROE), known for its high antioxidant activity, in soybean oil used to fry breaded butterfly shrimp, by measuring the induction period with OXIPRES, total polar material (TPM), peroxide index (PI), and free fatty acids (FFA). This evaluation was performed in comparison with control oils without antioxidants. The results showed a significant difference between the oils according to the analyzed parameters, especially in the final hours of frying. The treatment of the oil with rosemary extract effectively delayed its oxidation, having lower levels in all the oxidation markers that were analyzed. It was also found that rosemary extract is able to reduce oil consumption by fried foods. Therefore, ROE ensures soybean oil a high stability against oxidation and a longer shelf life, making it a good natural alternative to synthetic antioxidants.

## 1. Introduction

In the world, soybean is considered to be one of the most important sources of vegetable oils. It is the most widely consumed and extensively used in frying foods and other applications [1]. Since soybean oil contains polyunsaturated fatty acids, it has an excellent nutritional profile [1]. In spite of this, under heating conditions, it becomes unstable causing rapid quality deterioration through the oxidation process [2, 3]. As reported previously, throughout the frying process, vegetable oils such as soybean oil produce undesirable flavor compounds, and their oxidative stability changes due to hydrolysis, peroxidation, and polymerization [4]. These reactions are even more likely to happen due to repetitive use and heating of frying oils, especially in restaurants and food manufacturing facilities. Moreover, these oxidative reactions are also affected by other factors such as oxygen in the air, the humidity of food, temperature, and unsaturation of oils [5]. Oxidative reactions deteriorate the quality of the oil and affect the nutritional qualities of fried foods as well as their sensory characteristics [6, 7]. This deterioration is due to the absorption of oils by fried foods that increases with the duration of the frying process. Indeed, the more the oil oxidizes, the more viscous it becomes, and the more it is absorbed by the food [8, 9]. Therefore, several synthetic antioxidants including butylated hydroxyanisole (BHA), tert-butylhydroquinone (TBHQ), and butylated hydroxytoluene (BHT) were explored to extend the shelf life of frying oils, to delay the formation of undesirable components, and to improve the sensory properties of fried products [10]. However, it was shown in some studies that these substances were toxic and carcinogenic for humans [11]. Thus, their use was restricted, and some of them were even banned or used with specific legal limits recommended in Europe and the USA [12, 13]. In the meantime, extensive efforts were made to minimize or prevent vegetable oil oxidation through the development of safe and effective antioxidants [14]. In fact, in recent years, many studies have focused on rosemary extracts for their antioxidant activity and their ability to delay lipid oxidation, preserve food, and protect human health [15–17]. The main active compounds in rosemary responsible for this high antioxidant activity are carnosic acid, carnosol, and rosmarinic acid [4, 5]. Among the uses of rosemary extract, frying oils feature prominently because of the degraded fats generated during the frying process that requires its antioxidant action [3]. In several studies, rosemary extracts have proven effective in significantly reducing oxidation lipid levels [18, 19]. Additionally, they showed a significant effect on retarding color degradation and reducing rancidity by decreasing oxidative reaction level [20]. A recent study published by our laboratory showed a significant effect of antioxidant extracts of rosemary added to sunflower oil to frying chicken wings. Moreover, rosemary extracts improved the color and flavor of fried chicken wings [17]. Following these promising results, it is necessary to study the stabilization of soybean oil with rosemary extract (ROE) because of its vigorous use in food industries and its vulnerability to oxidation due to its high unsaturated fatty acid composition [2]. To date, the number of works that have studied the phenomenon of oil absorption using rosemary extract remains very limited; that is why our study evaluated the ability of ROE to reduce oil absorption during the frying of breaded butterfly shrimp, which are widely consumed worldwide, and especially when fried, they contain a large amount of water that evaporates during the frying process, where they absorb oil in return [21, 22]. The low oil content in a fried food is attractive to the consumer because this parameter can impact the taste and organoleptic and nutritional quality of the fried food. For these reasons, our research focused, using different oxidation markers, on the power of ROE to delay the frying oil oxidation and to reduce the oil content in fried foods.

## 2. Materials and Methods

### 2.1. Antioxidant Extraction

The extraction of the antioxidant from rosemary was performed according to the Robert and Georges Method (1989). Rosemary leaves were collected from natural plant populations in the Guercif region of Morocco and were extracted using hexane. This extract was then filtered, concentrated, and stirred with a sodium hydroxide solution. After the separation of the aqueous and organic phases, the solution was acidified with concentrated sulfuric acid and then extracted with dichloromethane to recover the antioxidant material. It was then added to an ethanolic extract and filtered from rosemary residue. The mixture was concentrated in a rotavapor and finally dried in a vacuum oven to prevent the deterioration of the antioxidants present. The extract obtained was analyzed by UHPLC Acquity H-class (Waters Company, Milford, MA, USA), with pulsed amperometric detection PAD over a measurement range of 284 nm, using analytical standards of carnosic acid and carnosol. The powder was diluted in vegetable sunflower oil to facilitate its incorporation into the frying oils. All chemical reagents used in the study were of analytical quality.

### 2.2. Accelerated Oxidation Test: OXIPRES

The performance of the obtained rosemary extract was evaluated by using OXIPRES (Mikrolab Aarhus A/S, Højbjerg, Denmark), to accelerate the oxidation process of soybean oil by exposing it to high temperature in an oxygen-rich environment. This allows for the attainment of high oxidation levels much faster than in real conditions. The oxidation of the sample is graphically recorded providing the induction period (IP). It is defined as the time elapsed from the test start to a decrease in oxygen pressure. The induction period, therefore, corresponds to the total oxidation or rancidity of the oil. The obtained IP can be converted into a predicted shelf life for the products tested [6]. The shelf life is calculated by Mikrolab Aarhus A/S using the following equation:

(1) $$\begin{array}{c} \text{Shelf life}=\text{IP x }A^{d}, \end{array}$$

where IP is the induction period, *A* is the acceleration factor (2), and *d* is the number of decades the analyzing temperature exceeds 20°C.

For this purpose, 1000 ppm of the rosemary extract was used to treat the soybean frying oil and which was then compared with the untreated sample (control). The aim was to evaluate the effectiveness of the rosemary antioxidant extract. The samples were analyzed in duplicate on two independent samples at a temperature of 90°C and a pressure of 5 bars.

### 2.3. Frying Oil and Shrimps

In this study, the soybean oil used was provided by Hala's brand. The peeled shrimp was purchased from a local market of Fez city, Morocco.

#### 2.3.1. Frying Oil Oxidation Kinetics

After preparing the extract of rosemary antioxidant (ROA), 800 ppm or 1000 ppm of this antioxidant was added to each sample of soybean oil. Then, they were heated to 190°C and used to perform 15 successive fryings per hour of breaded shrimp. Each cooking session requires 30 seconds, with 2 different shrimp/oil weight ratios of 1/80 and 1/100 until total oil oxidation. A control oil without treatment was performed under the same conditions.

As the frying process progressed, the quality of the oil was monitored to follow the evolution of the oil consumption and the oxidation compounds by measuring the following parameters: total polar material, free fatty acids, and peroxide index.

### 2.4. Total Polar Material (TPM)

The total polar material (TPM) represents all of the polar degradation products resulting from the frying process, including those from fried foods. In oil, they reflect the rate of deterioration and dissociation of its triglycerides. The analysis was carried out by a probe tester (Testo 270): deep-frying Oil Tester (Testo, Germany) according to the manufacturer's instructions. It can measure the dielectric constant variations in the oil, strongly correlated with the concentration of TPM, with an accuracy of ±2% of TPM [23]. According to French regulations, the maximum percentage of polar compounds allowed is 25%. Beyond this value, the oil is considered unfit for consumption [24].

### 2.5. Free Fatty Acids (FFA)

Free fatty acids (FFA), responsible for the acidity of the oil, are formed by thermal hydrolysis where the triglyceride molecule reacts with a water molecule to give an FFA and a diacylglycerol. This reaction occurs during frying due to the presence of water released from fried foods and the high temperatures used during this process [25]. FFA were used to characterize the degree of deterioration of frying oils. They are measured according to the official method of the American Oil Chemists' Society (AOCS Ca 5a-40, 2017) by neutralizing free fatty acids with 0.1 N of sodium hydroxide in the presence of ethanolic phenolphthalein as a color indicator. The maximum recommended value of FFA in frying oil is 0.9% and ideally ≤0.6% [26].

### 2.6. Peroxide Index (PI)

The peroxide index (PI) is used to evaluate the degree of primary oxidation in the frying oil. It quantifies the number of active oxygen molecules in the organic chains of fat that lead to the formation of hydroperoxides. These are responsible for the peroxidation of unsaturated fatty acids and gradually decompose into secondary oxidation products such as aldehydes and hydrocarbons responsible for rancid flavor and for bad taste [27]. The higher the index, the more fat material is oxidized [28]. The PI was determined according to the iodometric method (ISO 3960: 2017) which requires titration with a sodium thiosulfate solution of 0.01 N, of the iodine molecules released following the oxidation of iodides by the hydroperoxides of the solubilized oil in an acetic acid/isooctane mixture.

### 2.7. Oil Consumption

The absorption of oil by fried foods is a phenomenon often observed during any frying process. Different factors could cause this phenomenon: cooking temperature, the fried food/oil weight ratio, humidity, shape, and size of fried food [29]. Moreover, the oxidation of frying oil makes it very viscous and therefore easy to be absorbed by food [8, 30].

The oil consumption by the fried food was monitored at the end of the frying process by measuring the weight of the treated oil remaining and comparing it to that of the untreated oil.

### 2.8. Statistics

The OXIPRES analyses were performed in duplicate, the rest of the assays and measurements were performed in triplicates, and the results were expressed as mean value. Statistical data was processed in Statgraphics Centurion 16 software through the variance analysis (ANOVA) and the Tukey test (*p* < 0.05).

## 3. Results and Discussion

### 3.1. Composition of the Extract

Rosemary extract contains different compounds with antioxidant activity which can be obtained separately depending on the polarity of the solvents used during the extraction. The data obtained by UHPLC showed the presence of two major diterpenes, carnosic acid, and carnosol at 42% and 12%, respectively. This result was recently published by our laboratory [17].

### 3.2. Preliminary Evaluation of Antioxidant Activity: OXIPRES

The antioxidant activity of the obtained rosemary extract was determined in soybean oil by OXIPRES using the test conditions described above. The results obtained in Table 1 showed that there is a significant difference between the control sample and the treated oil. The time required for the total oxidation was higher in soybean oil added to rosemary extract. Embuscado [31] also used OXIPRES to evaluate the effect of rosemary and sage extracts on the stability of high oleic sunflower oil. Both extracts had interesting effects, especially when combined with other antioxidants such as citric acid and ascorbic acid [31]. The oils treated with rosemary and sage extracts had an IP of 13.2 hours and 9.8 hours, respectively, while the untreated control oil only took 5.4 hours. Another study conducted by Thomsen et al. [32] was aimed at reducing the rate of addition of vegetable oils to fish oils, used in pet food. In this study, rosemary extract and tocopherol were added to these mixtures, to check their oxidative stability using OXIPRES. The result showed that the addition of these antioxidants increases the IP of the fish oil which contains only 30% of vegetable oil.

### 3.3. Total Polar Material (TPM)

**Figure 1:**
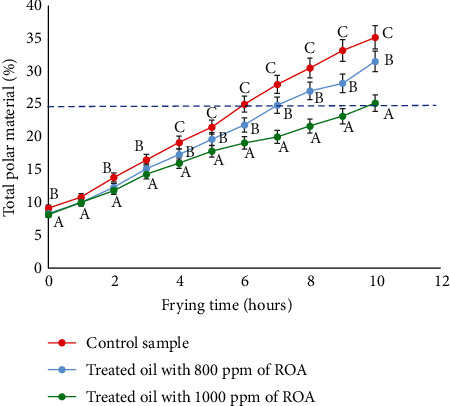
Formation of total polar materials during frying of breaded shrimps in soybean oil. The values followed by different letters (A, B, and C) are significantly different from each other at *p* < 0.05.

**Figure 2:**
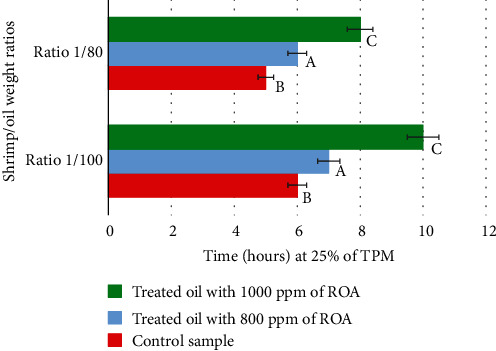
Effect of shrimp/oil weight ratios on the oil oxidation. The values followed by different letters (A, B, and C) are significantly different from each other at *p* < 0.05.

During frying, the measurement of the TPM was the main parameter to evaluate the stability of the oil. It is also the means to determine the duration of frying until the total oxidation of the oil, which represents 25% of TPM. The untreated control oil reached 25% of TPM after 6 hours of frying. The addition of 800 ppm of rosemary antioxidant seemed to have a slight effect against oxidation (Figure 1). This effect was improved by increasing the dose of ROA to 1000 ppm which made it possible to achieve 25% of TPM after 10 hours of frying. The results obtained are in accordance with those obtained previously in various studies. Among them, rosemary extract was demonstrated to have a significant effect on the polar compound formations and secondary oxidation. As compared to conventional synthetic antioxidants, these compounds enhanced soybean oil's oxidative stability by more than 30% [33]. Besides, Saoudi et al. [34] conducted a study on the incorporation effect of rosemary and thyme extracts on the oxidation prevention of soybean oil under heating and frying conditions for 24 h. The data showed that these extracts reduced the polar compounds by 70% compared to the control after 24 h of heating, and until the 15th fry, soybean oil improved considerably the overall acceptability of potato crisps. In terms of oxidative stability, rosemary extract had the strongest resistance until 6 hours after heating [34]. In a study conducted by Sayyad et al. [35], the thermoxidative stability of soybean oil was evaluated at 180°C for 20 h adding 3000 ppm of rosemary extract. It seems that the antioxidant rosemary reduced the total polar compounds more effectively than the synthetic antioxidant tert-butylhydroquinone (TBHQ). Similar results were obtained by Deora et al. [36] where rosemary extract showed superior efficacy to TBHQ in decreasing TPM and inhibiting palm oil degradation during noodle frying [36]. Furthermore, the effect of rosemary extract in frying oils, more specifically on decreasing TPM, was also demonstrated by Urbancic et al. [37]. In their study, rosemary extract was the most stable and efficient antioxidant agent compared to TBHQ, BHA, and tocopherols, after 20 deep-frying cycles. As reported, after 5 days of frying, oil with synthetic antioxidant TBHQ exceeded the TPM limit, whereas oil treated with rosemary extract could still keep the TPM below 25% [16]. The ratio of food/oil weights also has an impact on the oxidation of the frying oil. The higher the ratio, the faster the oxidation. This was illustrated in Figure 2 which consisted of evaluating the effect of the quantity of shrimp used compared to that of the oil on the oxidative stability during successive fryings. For this purpose, two shrimp/oil weight ratios were used: 1/80 and 1/100. The results showed that the time needed to reach a TPM of 25% was higher using the 1/100 ratio. Moreover, the alteration reactions are more intensive in both ratios of the control sample, and the rosemary extract delays the formation of polar compounds in both ratios. A study conducted by Rossell [29] proved that the optimum ratio of oil volume to the quantity of fried food keeps the frying temperature in an acceptable range.

### 3.4. Free Fatty Acids (FFA)

**Figure 3:**
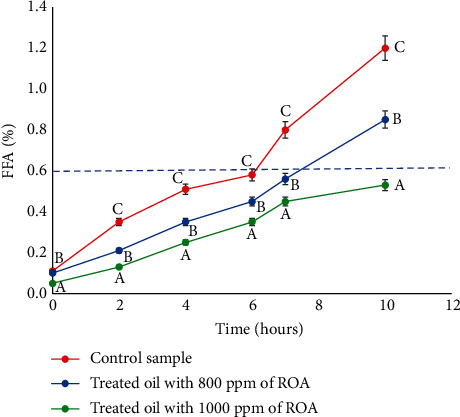
Development of free fatty acids during the frying of breaded shrimp in soybean oil. The values followed by different letters (A, B, and C) are significantly different from each other at *p* < 0.05.

In different countries and industries, FFA measurement is used as an indicator of oil deterioration due to the hydrolysis of triacylglycerol. In this experiment, FFA measurement was performed every two hours to evaluate the efficiency of the rosemary extract against the untreated control. It was observed that FFA increased with frying time for both treated and untreated oils. The rate of increase was lower for the oil containing rosemary extract at 800 ppm and even lower at 1000 ppm (Figure 3). An important increase in FFA was detected in untreated frying oil, which confirms that this oil was more susceptible to degradation during frying than the frying oils treated with an antioxidant of rosemary extract. It could be concluded that the addition of rosemary antioxidant effectively delayed the accumulation of FFA compared to the untreated control sample. In the topic of free fatty acids released in oils during the frying process, other studies obtained concurring results. Urbancic et al. showed that almost 50% of FFA formation in sunflower oil with added rosemary extract was inhibited after 20 cycles of deep-frying as compared to the control oil [37]. Further, the results obtained by Li et al. [16] reported that rosemary extract (0.02%) inhibited the soybean hydrolysis process by 26.19% after several times of frying and retarded the oxidation of the unsaturated fatty acids in a reduction in saturated fatty acid formation. Chammem et al. [38] studied the use of rosemary extract in a mixture of soybean and sunflower oil at 800 ppm to fry potatoes at 180°C. They demonstrated that the polyphenols present in this extract were able to delay the oxidation of oils that causes the release of free fatty acids [38]. A similar study on potatoes performed by Yıldırım [39] reported the effectiveness of rosemary extract on the reduction of FFA in sunflower oil at 2000 ppm. Similar results were obtained by Ammar (2016) who compared the effect of the rosemary antioxidant with a synthetic antioxidant (TBHQ) in palm oil used for frying beef meatballs. The data showed that rosemary extract had the superior ability to reduce the formation of FFA.

### 3.5. Peroxide Index (PI)

**Figure 4:**
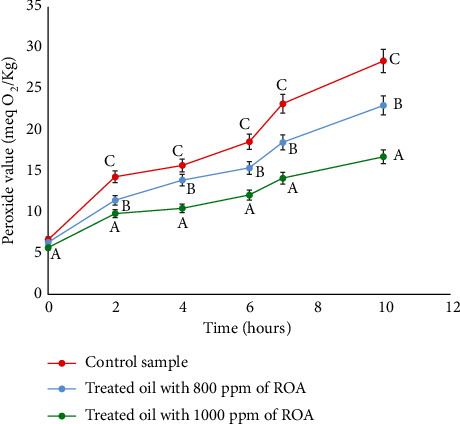
Development of peroxide index during the frying of breaded shrimps in soybean oil. The values followed by different letters (A, B, and C) are significantly different from each other at p <0.05.

The peroxide value is a standard measure widely used to evaluate the primary stages of lipid oxidation. A slower increase of PI implies higher oxidative stability [40].  Figure 4 shows that the addition of rosemary extract to soybean oil causes a significant decrease in PI compared to the control sample. After 10 hours of frying, the PI of the oils treated with rosemary extract at 800 and 1000 ppm reached maximum values of 23 and 16 meqO2/Kg, respectively, which were significantly lower than the control sample (28 meqO2/Kg). This clearly shows an inhibitory effect of rosemary extract against the oxidative rancidity of frying oil caused by hydroperoxides. These results are in agreement with those of Zhao et al. [41] who treated a mixture of soybean and palm oils with carnosic acid extracted from rosemary, carbon dots, and TBHQ. After 35 batches of fried meatballs, the oil with rosemary extract added had the lowest IP [41]. Yang et al. [42] did a study comparing rosemary extract to synthetic antioxidants (BHA and BHT) on soybean oil, which was stored in a proofer at 60°C for 24 days and analyzed every 6 days. Throughout the storage period, the PI of the oil treated with rosemary extract was significantly lower than that treated with synthetic antioxidants [42].

### 3.6. Oil Consumption

Monitoring and improving the amount of oil absorbed by fried foods are essential. This is the case, especially after several frying cycles where the oil becomes oxidized and contains harmful compounds that can be absorbed by fried foods and consequently consumed by humans [9].  Table 2 represents the evolution of soybean oil consumption after 10 hours of frying breaded shrimp with and without rosemary antioxidant. The use of 800 ppm of rosemary extract (ROE) reduces oil consumption compared to the control. However, this reduction is much greater when treating this oil with a higher dose (1000 ppm) of ROE. This could be explained by the ability of ROE to delay oil oxidation which is related to its consumption. It has been shown that the oxidation of the oil generates the appearance of short-chain fatty acids and volatile compounds responsible for the rancid note such as 2.4-heptadienal, 2-pentenal, 1-penten-3-ol, 2-butenal, propanal, and hexanal [43]. These volatile compounds continuously leave the oil due to the high temperature and steam during the frying process [44]. The nonvolatile compounds oxygenate, cyclize, polymerize, and gradually contribute to the increase of the viscosity of the oil [43]. All these discoveries can explain the large amount of oil consumed in the control. According to oxidation markers, untreated oil oxidizes faster than treated oil with rosemary extract (Table 2).

Therefore, faster formation of volatile compounds in the control oil and a higher viscosity enable its absorption by fried foods [9].

The results obtained are in concordance with those of Deora et al. [36] who monitored the consumption of palm olein oil during noodle frying. They noticed that noodles fried in oil with added rosemary extract had lower oil consumption than TBHQ and the control. Another study conducted by Tseng et al. [45] showed that the oil content on the surface of tortilla chips was higher in those fried in degraded oil than in fresh oil.

The moisture of the food and its oil absorption are two associated parameters, and a loss of moisture in fried products also means a high content of oil absorption because the steam escaped from the food leaves empty pores where the oil is penetrated [22, 46]. A study conducted by Yang et al. confirmed that oil uptake by shrimp increases with oil age during conventional frying processes. They also found that the moisture content of shrimp decreased by 30% after frying [21].

## 4. Conclusion

The present study revealed that rosemary extract (ROE) effectively slowed down the oxidation of soybean oil used for the frying of breaded butterfly shrimp. The oxidation markers used, TPM, FFA, and PI, were lower during the whole frying process in the soybean oil treated with rosemary extract as compared to the untreated control oil. This shows that the oxidation mechanisms stimulated during frying are slowed down by the addition of ROE. Rosemary extract also showed a high ability to reduce oil consumption by fried foods. The OXIPRES test confirmed that ROE was also able to extend the shelf life of frying oils. This finding underlines the economic interest and importance for vegetable oil producers who need to optimize their storage, as well as for the fried food industry, which consumes large quantities of oil. The impact of the ROE is also positive for the consumer through its beneficial effects on human health that will be passed by indirect consumption of the ROE present in the treated food or oil. ROE may therefore present a promising natural antioxidant source for the prevention of fried oils.

## Figures and Tables

**Table 1:** Induction period and predicted shelf life of the samples analyzed in OXIPRES.

Samples	IP at 90°C (hours)	IP at 20°C (hours)	Predicted shelf life (months)
Control	18.3	2342	3.3
Soybean oil treated with rosemary extract	24.3	3110	4.3

The IP at 20°C was calculated by Mikrolab Aarhus A/S.

**Table 2:** Comparative measurement of soybean oil consumption.

Experimental part	Control (%)	ROE 800 ppm (%)	ROE 1000 ppm (%)
Oil before frying	100	100	100
Oil at the end of frying (10 hours)	57	60	85
Total oil consumption	43	40	15

## Data Availability

Our results (figures and tables) are inserted in the text. References are included in the main text, and at the end of the manuscript, a list of references with their sources and DOIs is provided.
